# Prior Population Immunity Reduces the Expected Impact of CTL-Inducing Vaccines for Pandemic Influenza Control

**DOI:** 10.1371/journal.pone.0120138

**Published:** 2015-03-26

**Authors:** Kirsty J. Bolton, James M. McCaw, Lorena Brown, David Jackson, Katherine Kedzierska, Jodie McVernon

**Affiliations:** 1 School of Mathematical Sciences, University of Nottingham, Nottingham, United Kingdom; 2 School of Community Health Sciences, University of Nottingham, Nottingham, United Kingdom; 3 Vaccine and Immunisation Research Group, Melbourne School of Population and Global Health, University of Melbourne, Melbourne, Australia; 4 Murdoch Childrens Research Institute, Melbourne, Australia; 5 Department of Microbiology and Immunology, University of Melbourne, Melbourne, Australia; National Institute for Public Health and the Environment, NETHERLANDS

## Abstract

Vaccines that trigger an influenza-specific cytotoxic T cell (CTL) response may aid pandemic control by limiting the *transmission* of novel influenza A viruses (IAV). We consider interventions with hypothetical CTL-inducing vaccines in a range of epidemiologically plausible pandemic scenarios. We estimate the achievable reduction in the attack rate, and, by adopting a model linking epidemic progression to the emergence of IAV variants, the opportunity for antigenic drift. We demonstrate that CTL-inducing vaccines have limited utility for modifying population-level outcomes if influenza-specific T cells found widely in adults already suppress transmission and prove difficult to enhance. Administration of CTL-inducing vaccines that are efficacious in "influenza-experienced" and "influenza-naive" hosts can likely slow transmission sufficiently to mitigate a moderate IAV pandemic. However if neutralising cross-reactive antibody to an emerging IAV are common in influenza-experienced hosts, as for the swine-variant H3N2v, boosting CTL immunity may be ineffective at reducing population spread, indicating that CTL-inducing vaccines are best used against novel subtypes such as H7N9. Unless vaccines cannot readily suppress transmission from infected hosts with naive T cell pools, targeting influenza-naive hosts is preferable. Such strategies are of enhanced benefit if naive hosts are typically intensively mixing children and when a subset of experienced hosts have pre-existing neutralising cross-reactive antibody. We show that CTL-inducing vaccination campaigns may have greater power to suppress antigenic drift than previously suggested, and targeting adults may be the optimal strategy to achieve this when the vaccination campaign does not have the power to curtail the attack rate. Our results highlight the need to design interventions based on pre-existing cellular immunity and knowledge of the host determinants of vaccine efficacy, and provide a framework for assessing the performance requirements of high-impact CTL-inducing vaccines.

## Introduction

Producing the current generation of IAV vaccines—which reduce host susceptibility by inducing antibodies against viral surface proteins—requires antigenic characterisation of the IAV strain. Intensive global surveillance of seasonal strains enables predictions for the dominant IAV in the coming influenza season, facilitating the production of sub unit B cell vaccines with average efficacy of approximately 60 per cent in adults aged 18–65 [1]. However such vaccines exhibit lower efficacy in the elderly [2], may require two doses to be effective in children [3], and provide limited if any protection against pandemic viruses [4–7]. Furthermore, timely implementation of traditional vaccines developed specifically for newly emerging pandemic strains is unlikely to be achievable [8]. Vaccine stockpiles that protect against infection with, or transmission of, a broad set of IAV viruses remain desirable.

Prime-challenge experiments in animal models demonstrate that CTLs induced by exposure to a heterologous strain reduce viral loads and disease severity [9, 10]. Whilst experimental data on CTL responses in humans challenged with IAV is rare (but see [11, 12]), epidemiological studies have provided indirect evidence that CTL-immunity—mediated by antigenic-specific T cells directed toward conserved internal proteins [13–15]—reduces viral shedding [16, 17] and illness [17–19]. Collectively these studies hint that CTL responses, while highly unlikely to induce sterilising protection, decrease rates of onward transmission, and thus that enhancement of CTL responses with vaccines could suppress transmission of novel IAVs [20]. Understanding human cytotoxic T lymphocyte (CTL) responses to influenza, and the effect of these on disease pathogenesis, is key to developing an efficacious CTL-inducing vaccine and optimal strategies for its use.

Recent cohort and challenge studies have established that influenza-specific T cells that cross-react with pandemic and seasonal IAV are present in most adults without relevant strain-specific antibodies [12, 21–23]. CTL responses to pandemic, seasonal, and laboratory viruses are of similar prevalence and magnitude [22], and show little variation with birth cohort [24], consistent with immunological evidence that influenza-specific T cell immunity is broadly cross-reactive [25] and that CD8+ T cell epitopes are constrained by functional requirements [26, 27]. The prevalence of CTLs varies with age; children are unsurprisingly less likely to display T cell immunity to IAVs [28] and T cell immunity may also decline in elderly hosts [24].

Cellular responses to a novel IAV may differ between naïve and primed hosts [29], indicating that the epidemiology associated with naturally acquired CTL responses is a key consideration when exploring the impact of large-scale augmentation of cellular responses with vaccines. There is still uncertainty about the individual roles of CD4+ T cells [12], CD8+ T cells [30] and other cross-reactive responses [31], in mediating heterosubtypic IAV immunity and the functionality of widespread naturally acquired T cells in protecting against IAV in humans is unclear. Recently Sridhar *et al.* identified late effector cytokine secreting influenza-specific T cells as correlated with reduced severity of pH1N12009 infection [17]. Highly elevated levels of effector CTLs present following an acute infection may lead to sub-clinical infection severity [20]. Poly-cytokine producing CD8+ T cells—likely to have greater efficacy in controlling infections—are relatively rare, which may suggest the available CD8+ T cells are functionally limited [22]. Memory CD8+ T cells enable ready clonal expansion and may play a role in regulating influenza infections [32] particularly if they are abundant [33]. There is likely a rich distribution of potentially protective influenza-specific T cells across the population.

The host-level efficacy of CTL-inducing vaccines will likely depend sensitively on vaccine design. A number of vaccine formulations—including live attenuated vaccines, lipopeptide vaccines and viral vector vaccines—are able to induce influenza-specific T cells in vaccinated animals/humans. Experimental lipopeptide vaccines inducing virus-specific CTL responses in mice have been shown to provide a strong anti-viral effect, with IFN-*γ* producing T cells peaking around one week after vaccination, and the resulting memory CD8+ T cells long-lived and readily boosted upon challenge [34]. A corresponding reduction in viral load in vaccinated animals is associated with reduced morbidity and mortality [35], and findings in a related model system may be reasonably assumed to further constrain infection transmission [36]. Critically, there are indications that the ‘immunodomination’ of some epitope-specific CTLs by those directed toward other epitopes may be misaligned with the relative virus clearing capacities of these cells, suggesting that naturally induced CTL responses may not realise the full potential of CTLs to control infection [37, 38]. A major complication in developing these vaccines for use in humans is the large number of MHC class I haplotypes each recognising different T cell epitopes, making high levels of effective vaccine coverage a challenge at a population level [20]. Current vaccine approaches include development of multi-epitope vaccines targeted towards the six most common HLA supertype families, which collectively represent <85 per cent of the population [35].

Live attenuated influenza vaccines trigger the production of influenza-specific T cells in children, but may not increase numbers of influenza-specific T cells in adults [39], indicating antigen presentation as well as prior exposure may influence vaccine efficacy [40]. If a vaccine cannot significantly boost cellular protection in hosts with pre-existing influenza-specific T cells, it may have greater utility for priming naïve CTL precursors in children. In contrast, strain-specific sub-unit B cell vaccines developed for pandemic influenza can show reduced efficacy in young hosts [41].

Viral vector vaccines—using recombinant viruses expressing NP and M1 IAV genes—have been shown to induce influenza-specific CD4+ and CD8+ T cell responses in adults of all ages [42, 43] that reduce disease severity [44]. Viral vector vaccines can induce protective responses that last in excess of 10 months in mice [45]. The efficacy of this class of vaccines in children is not yet known. Vaccines able to boost CTL responses in hosts with cross-reactive memory CD8+ T cells may show broad efficacy across age ranges, and may also have utility in preventing T cell immunosenescence in elderly hosts [43].

Traditionally models capturing influenza transmission have not been configured to account for population heterogeneities in cellular immunity and thus have limited utility to predict the impact of a realistic candidate CTL-inducing vaccine. Recently Arinaminpathy *et al.* considered the impact of widespread distribution of a partially protective vaccine targeting conserved antigen on pandemic attack rates and the turnover of seasonal influenza strains [46]. The adopted model tracks the emergence of new clusters of antigenic variants of a single influenza subtype. Infection by a strain in one cluster confers partial protection against strains in another depending on their phenotypic relationship [47], thus capturing the influence of partially protective cross-reactive antibody. However the model does not incorporate the heterologous cross-protective effects that may be offered by CTLs induced via natural infection, or consider the possibility that vaccine efficacy is heterogeneous with host infection history.

Models for the infection dynamics of pandemic IAV must consider the potential for pre-existing cellular and humoral immunity to the pandemic virus in the population, which may vary considerably between candidate pandemic IAVs. Cross-reactive antibodies to pH1N12009 detectable via haemagglutination inhibition assays were only found in elderly hosts prior to pandemic onset in 2009 [48]. In contrast, the pH1N12009 T-cell epitopes were highly homologous with those of recently circulating seasonal viruses [13] and cross-reactive T cells common in adults [22]. However at least one of the immunodominant peptides for pH1N12009 was different to those expressed by cells infected with IAV viruses circulating in the last 60–90 years [14], which may have contributed to the ability of this virus to trigger an outbreak of pandemic proportions. Recently an H3N2 IAV of swine origin (H3N2v) caused over 300 confirmed cases of influenza in the US. Instances of human-human transmission have been reported, and although there were likely many undetected cases, transmission was not sustained [49]. Characterisation of the relationship of the T cell epitopes of H3N2v to those of recently and historically circulating influenza suggests that the NP and M proteins of H3N2v are closely related to those in seasonal viruses circulating prior to 1950. However H3N2v is also antigenically related to a human H3N2 virus from the 1990’s that has been circulating in swine for a number of years [50], and indeed seroprevalance of cross-reactive antibodies is high amongst adults (McVernon, CDC conference, 2013). Other viruses with pandemic potential such as avian H5N1 or H7N9 IAVs, subtypes that have not circulated widely in humans over the past generation, are likely to induce cross-reactive cellular responses [51], but are unlikely to be recognised by neutralising antibody [52].

In this paper we explore the uncertainties in the predicted impact of CTL-inducing vaccines on the spread of a novel virus, given uncertainties in the strength of the cross-protection afforded by influenza-specific T cells triggered by infection with seasonal IAV variants, the prevalence of humoral immunity and the (immune state dependent) efficacy of a CTL-inducing vaccine. We compare the impact of a hypothetical CTL-inducing vaccine administered to a fully naïve population, to the impact in a population in which some hosts have a primed CTL response or cross-reactive neutralising antibody. In addition, we use a simple phenomenological model to estimate the opportunity for drift variants to emerge during the pandemic to explore the potential impact of CTL vaccination campaigns on the antigenic evolution of IAV.

## Materials and Methods

### Infection dynamics of pandemic IAV

**Fig 1:**
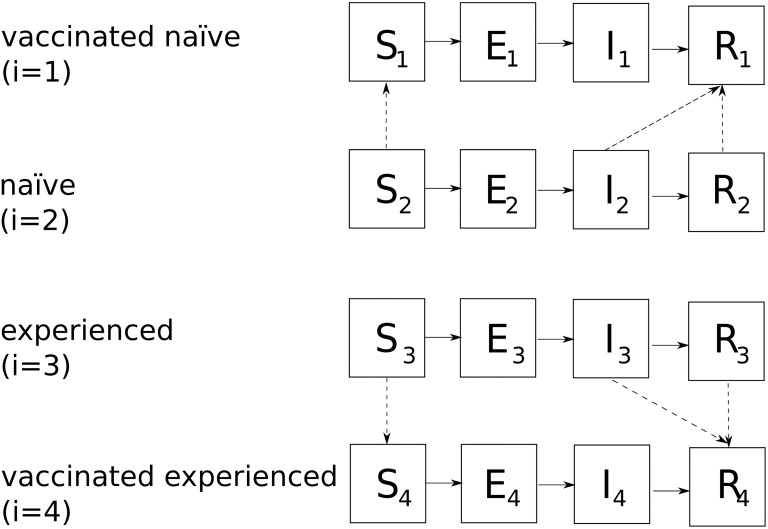
Model structure. Susceptible hosts are divided into 4 strata (labelled by *i*) depending on their prior influenza experience and vaccination status. Susceptible hosts *S*_*i*_ become exposed (*E*_*i*_) at a rate proportional to *β*_*f*_, become infectious (*I*_*i*_) with Erlang distributed waiting time with rate parameter *γ* = 1/(1.3 days) and recover (*R*_*i*_) at a rate *ν* = 1/(1.6 days). Dashed lines indicate the vaccination of hosts. Note we assume that vaccines do not alter the infectiousness of already exposed hosts (although this distinction is of little consequence if vaccination occurs very early in a pandemic). We allow for the possibility of strain-specific antibody to the pandemic strain by assuming that some (experienced) hosts begin in the recovered state *R*_3_. We assume infected-acquired immunity is maintained over the course of the simulation, however in practice as immunity wanes recovered hosts who were originally naïve migrate to the appropriate *experienced* susceptible state (*R*_1_ → *R*_4_, *R*_2_ → *R*_3_).

We construct a minimal model to perform scenario analyses for assessing the potential impact of CTL-inducing vaccines in a static population with both pre-existing cellular and humoral immunity to a pandemic virus which has structure as shown in Fig 1. In our model CTL-inducing vaccines reduce the potential infectiousness of vaccinated hosts in a manner that depends on pre-existing host immunity.

Our model has 4 main classes of hosts, each occupying an *SEIR* stratum; vaccinated naïve (*i* = 1), unvaccinated naïve (*i* = 2), unvaccinated experienced (*i* = 3), and vaccinated experienced (*i* = 4). Susceptible hosts become exposed at a rate governed by *β*_*f*_ and move through a latent state *E* (implemented using the method of stages to provide Erlang-distributed waiting times from contact to infection [53, 54]). We assume that exposed hosts become infectious *I* on a time-scale of *γ* ≡ 1/*T*_*e*_ = 1/(1.3 days) (in keeping with recent estimates of the latency period [55, 56]), and recover to *R* at a rate *ν* ≡ 1/*T*_*i*_ = 1/(1.6 days), as described below:

$$\begin{array}{ccc} \dot{S_{i}} & = & -\sum_{j}S_{i}I_{j}\beta_{ij},\\ \dot{E_{i}} & = & \sum_{j}S_{i}I_{j}\beta_{ij}-2\gamma E_{i}\\ \dot{E_{i}^{\prime }} & = & 2\gamma (E_{i}-E_{i}^{\prime })\\ \dot{I_{i}} & = & 2\gamma E_{i}^{\prime }-\nu I_{i},\\ \dot{R_{i}} & = & \nu I_{i},\\ \beta_{ij} & = & \beta_{f}(\begin{array}{cccc} \epsilon_{N}m_{NN} & m_{NN} & \epsilon m_{NE} & \epsilon_{E}m_{NE}\\ \epsilon_{N}m_{NN} & m_{NN} & \epsilon m_{NE} & \epsilon_{E}m_{NE}\\ \epsilon_{N}m_{EN} & m_{EN} & \epsilon m_{EE} & \epsilon_{E}m_{EE}\\ \epsilon_{N}m_{EN} & m_{EN} & \epsilon m_{EE} & \epsilon_{E}m_{EE} \end{array})\cdot \end{array}$$ (1)

where *ϵ*_*N*_, *ϵ*, *ϵ*_*E*_, ∈ [0, 1] respectively denote the infectiousness of vaccinated naïve, unvaccinated experienced and vaccinated experienced hosts relative to unvaccinated naïve hosts. Variables are expressed in numbers of hosts unless otherwise specified. Our choice for *T*_*e*_ and *T*_*i*_ yields a generation time between successive cases of 2.9 days, consistent with average estimates from pH1N12009 surveillance data [57–59], however our results are not altered when adopting other plausible choices for *T*_*e*_ and *T*_*i*_.

We assume that a fraction *f*_*E*_ of hosts are “influenza-experienced”; having had a previous infection with IAV that confers cross-reactive cellular, and potentially also cross-reactive humoral, immunity to the pandemic virus. The remaining hosts are denoted “influenza-naïve”. The strength of the cellular immunity amongst influenza-experienced hosts *ϵ* and the prevalence of the humoral immunity amongst influenza-experienced hosts are specified independently of *f*_*E*_ (see below). With the simplifying assumption that lifetime risk of infection increases with age, our choice of *f*_*E*_ = 0.8 roughly translates to assuming that hosts older than 15 have had an infection with IAV (assuming age-demographics for an industrialised country such as Australia, see *e.g.* http://www.abs.gov.au/). Alternate values for *f*_*E*_ are explored in a sensitivity analysis. The population size *N* is nominally chosen to be 1 million.

We explore results assuming homogeneous/random mixing (*m*_*NN*_ = *m*_*EE*_ = *m*_*NE*_ = *m*_*EN*_ = 1) or weakly assortative mixing with *m*_*EE*_ = 0.7, *m*_*NN*_ = 1.4, *m*_*EN*_ = *m*_*NE*_ = 1. The latter choice mimics the increased contact rate between younger hosts and between adults and younger hosts seen in survey data [60, 61].

### Vaccination

In the absence of firm knowledge of the immunological determinants of host response to a CTL-inducing vaccine, and the epidemiology associated with CTL-immunity, we explore a range of assumptions for prior immunity and vaccine action. In particular, we consider a scenario in which the population has no prior cellular or humoral immunity (A). Such an assumption is likely appropriate if the pandemic strain is of novel subtype and if influenza-specific T-cells commonly found in the population are alone insufficient to suppress transmission from infected hosts (perhaps because elevated levels of effector T cells are required [20]). We also consider three scenarios (B–D) in which influenza-experienced hosts have pre-existing cellular, and perhaps also humoral/antibody, immunity. In scenarios B and C we assume that influenza-experienced hosts have CTL-mediated protection resulting in infectiousness that is 50 per cent that of naïve hosts (*ϵ* = 0.5), but consider alternative vaccine efficacy assumptions in experienced hosts between the two scenarios (see below). Such protection may reflect the distribution of effective cellular immunity if the influenza-specific T cells found in most adults [12, 21–23] reduce viral shedding. The fourth scenario (D) is motivated by the emergence of H3N2v—a virus with HA protein recognised by antibodies in many adult hosts (McVernon, CDC conference, 2013)—and assigns neutralising antibody to 50 per cent of influenza-experienced hosts in addition to their reduced infectiousness due to cellular immunity (*ϵ* = 0.5).

Initially all states are empty except for *S*_2_ = (1 − *f*_*E*_)*N* and *S*_3_ = *f*_*E*_ *N* (Scenarios A–C). Hosts with pre-pandemic sterilising immunity begin in the compartment *R*_3_ in our model, yielding initial conditions *S*_2_ = (1 − *f*_*E*_)*N*, *S*_3_ = *f*_*E*_ *N*/2 and *R*_3_ = *f*_*E*_ *N*/2 for scenario D. Variations on these scenarios are explored in the sensitivity analysis.

We denote the infectiousness of vaccinated naïve hosts relative to unvaccinated naïve hosts *ϵ*_v_. In scenario A, when there is no pre-pandemic immunity, the infectiousness of all vaccinated hosts is uniform. In scenario B we consider a ‘saturating’ vaccine that may not boost existing CTL responses (*ϵ*_*N*_ = *ϵ*_v_, *ϵ*_*E*_ = min(*ϵ*, *ϵ*_v_)). Such vaccine action may be representative of the action of live influenza vaccines able to induce T-cell responses in children more readily than in adults [39]. In scenarios C and D we consider the impact of a second class of vaccine; a ‘boosting’ vaccine that enhances protection against transmission in experienced hosts who already have naturally acquired CTL protection by the same factor as for naïve hosts (*i.e.* *ϵ*_*N*_ = *ϵ*_v_, *ϵ*_*E*_ = *ϵϵ*_v_). Viral vector vaccines that show promising results in adults may have such an action if they prove efficacious in children. Lipopeptide vaccines may also have the potential to boost CTL immunity in hosts with a range of pre-existing naïve and memory CTL pools, depending on their design [20]. Table 1 summarises the scenarios A–D examined in the main text.

**Table 1:** Scenarios. The infectiousness of vaccinated and/or experienced hosts (relative to unvaccinated naïve hosts) for the four scenarios A–D presented in the main text. *ϵ*_v_ is a free parameter that is set to determine the infectiousness of vaccinated naïve hosts relative to unvaccinated naïve hosts.

Scenario	Prior cellular immunity (relative infectiousness = *ϵ*)	Prior humoral (sterilizing) immunity	Vaccine	Vaccinated naïve infectiousness (*ϵ*_*N*_)	Unvaccinated experienced infectiousness (≡ *ϵ*)	Vaccinated experienced infectiousness (*ϵ*_*E*_)
A	None	None	Uniform (vaccine boosts proection uniformly due to fully susceptible population)	*ϵ*_v_	–	–
B	80% of population	None	Saturating vaccine	*ϵ*_v_	*ϵ*	min(*ϵ*, *ϵ*_v_)
C	80% of population	None	Boosting vaccine	*ϵ*_v_	*ϵ*	*ϵ* × *ϵ*_v_
D	80% of population	50% of experienced population	Boosting vaccine	*ϵ*_v_	*ϵ*	*ϵ* × *ϵ*_v_

For simplicity we assume that vaccine protection lasts for the duration of the simulation. We explore rapid vaccine distribution to the general population early in the pandemic (with baseline assumption that vaccines are distributed over 10 days) at a range of coverage levels, or pre-emptive targeted distribution of a stockpile allowing 50 per cent coverage. As vaccines inducing T cell responses are still in the design phase, we explore the full range of possible values for *ϵ*_v_ ([0, 1]). This parameter can be considered to subsume both ‘uptake’ (vaccinated proportion) and ‘take’ (effectively immunised proportion) given the anticipation of only partial population coverage, resulting from HLA class restriction of CTL epitopes.

### Effective reproduction number and the attack rate

The value of the basic reproduction number *R*_0_—indicating the average number of transmitted infections per infected host—expected for future pandemic outbreaks is uncertain. Inferences using mortality and morbidity data from the 1918 Spanish influenza pandemic suggest that *R*_0_ may be as high as 4–6 [62]. The measured effective reproduction number for that pandemic—a composite parameter recognising the constraining influence of the underlying immune profile, social mixing patterns and variations in surveillance strategies—was estimated to be much lower, of order 1.3–3 [63]. Estimates of the effective reproduction number for pH1N12009 [64], and the first wave of the 1968 pandemic [65], also range between 1 and 2.

We consider a pandemic virus that at pandemic onset and prior to vaccination spreads at a moderate rate *R*_eff_ = 2. At the time of emergence of a pandemic virus, the existing immune status of a given population is unknown, allowing estimation of *R*_eff_ but not *R*_0_ from early observations of the epidemic growth rate [66]. For this reason, in our main analysis we have constrained all studied simulations to have a uniform *R*_eff_ = 2 prior to intervention, enabling us to consider the influence of underlying prior immunity on vaccine impact for epidemics that may otherwise appear the same. We explore more severe pandemic scenarios, and a scenario in which the final size of the epidemic, rather than the initial growth rate, is held fixed in the sensitivity analysis. *R*_eff_ depends on the population susceptibility profile (including *ϵ* and *f*_*E*_) and is the maximum eigenvalue, *M*, of the matrix constructed by multiplying each row *i* of *β*_*ij*_ (Equation 1) by *S*_*i*_(*t*). In scenario A the absence of any pre-pandemic immunity renders the basic and effective reproduction numbers equal (*i.e.* *R*_0_ = *R*_eff_). To fix *R*_eff_ between scenarios the factor *b*_*f*_ must vary. If there is pre-existing cellular or humoral immunity, *R*_0_>*R*_eff_ and *β*_*f*_ is higher than for a scenario without prior immunity. Assuming homogeneous mixing *β*_*f*_ = *R*_eff_/(*NT*_*i*_) for scenario A, *β*_*f*_ = 5*R*_eff_/(3*NT*_*i*_) for scenarios B & C, and *β*_*f*_ = 5*R*_eff_/(2*NT*_*i*_) for scenario D at baseline (*i.e.* unimpeded by vaccination).

The proportion of the population infected over the course of an outbreak is known as the ‘final epidemic size’ or ‘attack rate’ (despite not being a ‘rate’). We adopt the latter terminology here. We evaluate it numerically as lim_*t* → ∞_∑_*i*_ *R*_*i*_(*t*) or equivalently, $\nu \sum_{i}\int_{0}^{\infty }I_{i}(t)dt$.

### Antigenic drift

Changes in the pandemic virus may have been responsible for the increased severity of the second wave of the 1968–9 H3N2 pandemic [67]. Changes in the dominant circulating virus between successive waves of pH1N12009, although not antigenically significant, were also noted [68]. Vaccination campaigns, by altering the immune landscape, have the potential to accelerate, or at least influence, IAV evolution. Modelling frameworks enabling estimation or prediction for the propensity for viral evolution during an epidemic are thus valuable. Invasion by a phenotypically novel variant is an inherently stochastic process more likely to occur in the genetic bottleneck between epidemic waves [69] which we cannot simulate in our single strain deterministic model. However we may estimate the opportunity for generation of new antigenic types and the selection advantage they may be afforded.

To estimate the opportunity for antigenic drift, we assume that each infectious host in each stratum *I*_*i*_ has the potential to transmit a new variant. We calculate the “drift opportunity” *P*_*emg*_—the cumulative opportunity for drift variants to emerge over the course of the epidemic—by integrating over all infections as below:

$$\begin{array}{c} P_{\mathrm{emg}}=\frac{1}{N}\int_{t=t_{e}}^{t_{\mathrm{end}}}dt\;\sum_{i}I_{i}\left(t\right)g_{0,i}{\left(t-t_{e}\right)}^{1\cdot 5}, \end{array}$$ (2)

where *t*_end_ corresponds to the resolution of the epidemic. Note that we only report dimensionless values of *P*_emg_ that are normalised by the value for the same epidemic without intervention. *P*_emg_ > 1 is possible and indicates an epidemic in which drift opportunity under intervention was greater than than in the absence of intervention. The non-linear dependence on time in Equation (2) which allows for *P*_emg_ > 1 is motivated by results from theoretical models for antigenic drift suggesting that new variants may be more likely to circulate later after introduction of the initial strain [70, 71]. The adopted power-law dependence on time is a phenomenological fit to the rate of antigenic cluster turnover for H3N2 from Koelle *et al.* [47]. We have assumed that *t*_*e*_ is the time since the cumulative incidence reaches 1 (host) in our deterministic model.

The factor *g*_0,*i*_ in Equation (2) is the per infection rate of emergence of new variants for hosts in stratum *i*. Arinaminpathy *et al.* assume that the per infection rate generating new variants depends only on cluster age [46] (*i.e.* *g*_0,*i*_ = 1). However, the emergence and thus transmission of variants from a host may be more common when infections are prolonged and viral replication occurs in the presence of sterilising or cross-reactive antibody [72, 73]. Prime-challenge experiments in macaques indicate that priming with seasonal H1N1 viruses 4 months prior to challenge with pH1N12009 enables CTL responses that clear the infection before appearance of neutralising antibody [74], suggesting that infections in vaccinated hosts with a readily primed CTL response may be associated with a significantly lower probability of selecting and transmitting antigenically novel variants. We thus explore 3 forms for the contribution of each infection to the overall probability of antigenic emergence: (i) *g*_0,*i*_ = 1 (per infection rate of emergence of new variants independent of vaccination and pre-pandemic immune status), (ii) *g*_0,*i*_ = *ϵ*_*i*_ (per infection rate of emergence of new variants proportional to net viral load/host infectiousness), and (iii) the distribution *g*_0,1_ = *g*_0,2_ = *g*_0,3_ = *ϵ*_*i*_, *g*_0,4_ = 0 (per infection contribution equal for all hosts except no contribution from CTL-vaccinated experienced hosts). Here *ϵ*_*i*_ is the infectiousness of hosts in the *i*th stratum. It is also possible in principle that CTL responses may enhance selection of novel variants, in which case scenarios with *g*_0,*i*_ > 1 may be justified. Such a possibility is not explored in this work.

Antigenic variants may have mutations that confer increased virulence/transmissibility and/or escape recognition of antibodies to the original pandemic virus and thus facilitate the process of antigenic drift. We focus on the selective advantage of the latter type of antigenic variant, assuming that the intrinsic transmissibility is unchanged from that of the original pandemic strain. The selective advantage of a new variant is largely governed by the ratio of effective reproduction numbers for the replacing and resident strains [75]. To gauge the probability that an antigenic variant that emerged during the pandemic (with probability *proportional* to our calculated “drift opportunity”) is selected, we consider this ratio at the conclusion of the pandemic. The effective reproduction number of the emerging strain *R*_eff,new_ is calculated assuming that it escapes all antibody-mediated immunity remnant after resolution of the initial pandemic wave. The ratio *R*_eff,new_/*R*_eff_ naturally increases with the prevalence of immunity to the resident pandemic strain.

## Results

### Mitigating potential of CTL-inducing vaccines

**Fig 2:**
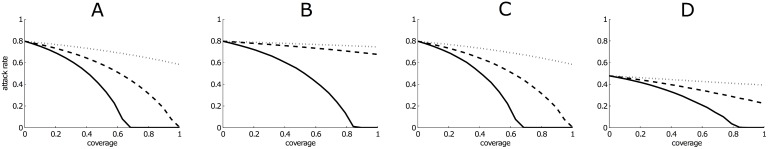
Attack rate with coverage for scenarios A-D. Predicted attack rates for four different pandemic and vaccine action scenarios as a function of general population coverage with a CTL-inducing vaccine. In each panel the solid line corresponds to assumptions of relative infectiousness in vaccinated and unvaccinated naïve hosts of *ϵ*_v_ = 0.25, with dashed and dotted lines corresponding to *ϵ*_v_ = 0.5 and 0.75 respectively. Scenario A captures a population without pre-existing CTL-mediated immunity to the pandemic strain. In scenarios B and C, 80 per cent of the population have pre-existing CTL-mediated immunity (*f*_*E*_ = 0.8) suppressing infectiousness by 50 per cent (*ϵ* = 0.5), with vaccine action saturating (B) or boosting (C). Scenario D is a modification of scenario C that assumes 50 per cent of those with naturally acquired CTL immunity also have cross-reactive protective antibody.

Our predictions for the attack rate as a function of CTL-inducing vaccine coverage vary significantly with underlying population immunity and vaccine action for pandemic scenarios A–D with the same initial growth rate. The mitigating potential is very similar for homogeneous and assortative mixing scenarios when vaccines are distributed uniformly amongst the population (see Fig 2 and S1 Fig).

In this subsection we focus our discussion on the homogeneous mixing case (Fig 2) but highlight interesting differences to the assortative mixing case. In the absence of any prior immunity (scenario A) the impact of vaccination is simple to predict (see [46]). The highest efficacy vaccine considered, which suppresses the infectiousness of vaccinated naïve hosts to *ϵ*_v_ = 0.25 relative to unvaccinated naïve hosts, can mitigate the pandemic with 65 per cent coverage. Attack rates are approximately halved to 40 per cent if coverage with such a vaccine is 50 per cent.

For scenarios in which there is only prior cellular immunity to the pandemic strain (B & C) baseline attack rates are equal to that for scenario A for fixed *R*_eff_. Note that this is only strictly true when mixing is homogeneous as the introduction of assortative mixing influences the value of *R*_0_ for fixed *R*_eff_, however the variation between scenarios A–C is very small for our choice of assortative mixing (see S1 Fig). In scenario B, unless the ability of the vaccine to suppress infectiousness is superior to the suppression arising due to naturally acquired pre-existing CTL immunity, even high levels of vaccine coverage will not significantly reduce the attack rate. In particular, attack rates would still be above 70 per cent if coverage of 50 per cent were achieved for vaccines with *ϵ*_v_ ≳ 0.5. The benefit of widespread vaccination in scenario B is only mildly inferior to that for scenario A when *ϵ*_v_ = 0.25. In contrast, if the vaccine can further suppress the infectiousness of vaccinated experienced hosts (scenario C), predictions for vaccine impact are more promising. Indeed the mitigating impact of vaccination campaigns achievable in scenario A is recovered.

In scenario D the overall attack rate is lower than scenarios A–C despite assuming the same initial growth rate, as the size of the cohort without protective antibodies is limited to 0.6*N*. Baseline attack rates amongst the naïve subpopulation however are equivalent to those in scenarios A–C when mixing is random (S2 Fig). As a larger portion of the initial growth rate is driven by the naïve hosts, such individuals are preferentially infected driving *R*_eff_ below 1 more rapidly than in the other scenarios considered. The accuracy of predictions for the attack rate from early estimates of *R*_eff_ is thus clearly contingent on understanding underlying population heterogeneities in mixing and immunity. Despite the lower attack rate in scenario D, overall attack rates decline more slowly with coverage than for the other scenarios that assume a boosting vaccine (*i.e.* scenarios A & C, see Fig 2). Slightly higher coverage with the most efficacious vaccine considered (*ϵ*_v_ = 0.25) is required to halt the pandemic than for scenarios A or C, as many vaccines are ‘wasted’ preventing transmission from hosts who are not susceptible (Fig 2). Distributing CTL-inducing vaccines to the general population will have greater power to reduce epidemic spread when there is little existing neutralising cross-reactive antibody to the pandemic strain.

### Distribution strategies for CTL-inducing vaccines

**Fig 3:**
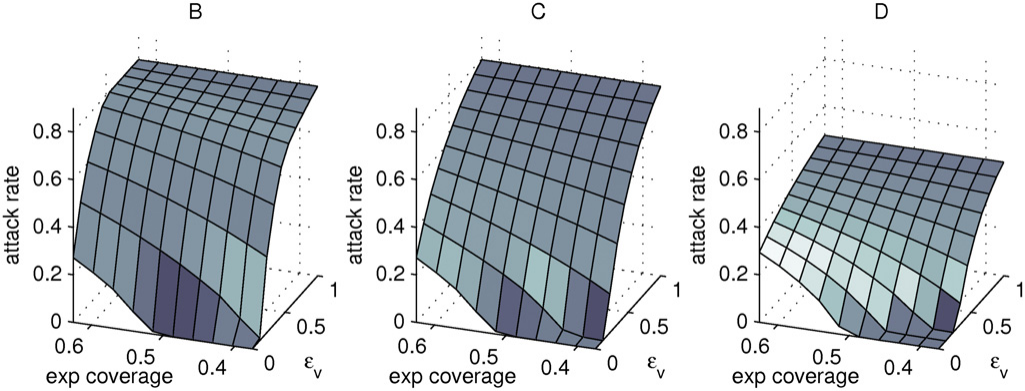
Attack rates for targeted vaccination in Scenario C. Epidemic attack rates for targeted distribution of a vaccine stockpile sufficient for 50 per cent coverage. For all epidemics the initial effective reproduction number in the absence of vaccination is fixed at 2. The axis labelled “exp coverage” denotes coverage in the experienced population and *ϵ*_v_ is the relative infectiousness of vaccinated and unvaccinated naïve hosts. The minimum plotted value of “exp coverage” indicates full coverage in the naïve population with the remainder given to experienced hosts, the maximum plotted value is for a scenario in which all vaccines are given to experienced hosts, and a value of 0.5 indicates equal coverage in naïve and experienced hosts (*i.e.* general population distribution). Panels represent estimates for different pre-pandemic immunity and vaccine action scenarios; 80 per cent of the population with naturally acquired CTL-mediated immunity (*f*_*E*_ = 0.8) and saturating vaccine action (scenario B), the same population with a vaccine that boosts protection in all hosts (scenario C), a population in which 50 per cent of those with naturally acquired CTL responses also have protective antibody to the pandemic strain and vaccine action is again boosting (scenario D). The strength of naturally acquired CTL-mediated immunity is set to *ϵ* = 0.5.

We explore the impact of distributing vaccines to varying proportions of naïve and experienced hosts assuming both homogeneous and assortative mixing for scenarios B–D (*i.e.* scenarios which assume heterogeneous pre-pandemic immunity). The impact of this targeted distribution when mixing is homogeneous is depicted in Fig 3. The advantage of distributing vaccines to naïve hosts in scenario B is intuitive when *ϵ*_v_ ≳ *ϵ* and the vaccine cannot reduce the potential infectiousness of experienced hosts. However, reduced attack rates when targeting naïve hosts are apparent even when the vaccine suppresses the transmission potential by the same factor in naïve and experienced hosts (scenarios C & D). Assuming *R*_eff_ = 2 and a vaccine stockpile sufficient for 50 per cent population coverage, distribution of a vaccine that completely blocks transmission (*ϵ*_v_ = 0) won’t halt population spread unless ≳50 per cent of naïve hosts are vaccinated in scenarios B-D (Fig 3). It is important to note that the preference for vaccinating naïve hosts in Fig 3 is purely driven by their larger potential infectiousness. The optimal efficiency of such ‘equalising’ vaccine strategies have also been discussed elsewhere (see, for example, [76]). As a result, the benefit of targeting naïve hosts (*i.e.* small values of “exp coverage”) shows a similar trend across scenarios B–D (Fig 3), but with some important differences. In particular, such targeted strategies enable mitigation with poorer performing, but still efficacious (*ϵ*_v_ < 1), vaccines.

When assuming random mixing, targeted vaccination in scenarios B & C does not alter the relative attack rates in naïve and experienced hosts, as all hosts are equally susceptible. However targeting naïve hosts in scenario D can markedly reduce the attack rate in this sub-population; the attack rate could potentially be constrained to below 10 per cent if *ϵ*_v_ ⪅ 0.35. In contrast, targeting experienced hosts in scenario D would enable widespread infection of naïve hosts with sub-population attack rates in excess of 50 per cent if *ϵ*_v_ ≳ 0.5 (data not shown). Adopting assortative mixing (as described earlier) instead of homogeneous mixing also increases the predicted benefit of reducing the coverage amongst influenza-experienced hosts for low to intermediate values of *ϵ*_v_ (S3 and S4 Figs).

### CTL-inducing vaccines and antigenic drift

**Fig 4:**
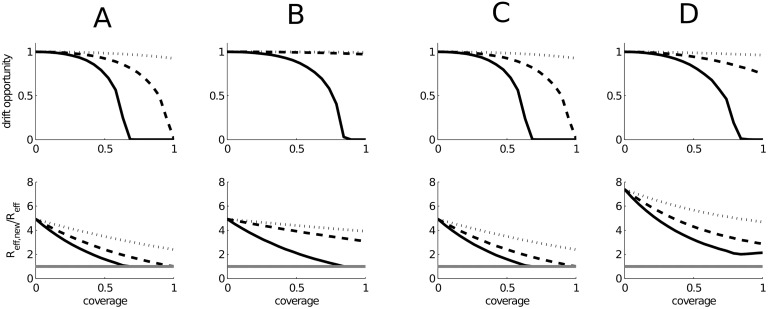
Drift opportunity with coverage for scenarios A-D. **Upper panels:** drift opportunity relative to an unimpeded epidemic as a function of general population coverage for four different pandemic and vaccine action scenarios assuming *R*_eff_ = 2, homogeneous mixing and *g*_0,*i*_ = 1. **Lower panels:** The ratio of effective reproduction numbers of antigenically novel and resident pandemic variants. The grey line indicates a ratio of unity. In each panel the solid line indicates results assuming that the relative infectiousness in vaccinated naïve and naïve hosts is *ϵ*_v_ = 0.25, with dashed and dotted lines *ϵ*_v_ = 0.5 and 0.75 respectively.

For our baseline parameter assumptions, with *R*_eff_ = 2, the drift opportunity *P*_emg_ decreases with coverage and is necessarily null when the pandemic is mitigated. However, when the per infection probability of the emergence of variants is constant (*i.e.* *g*_0,*i*_ = 1)—as assumed in previously published work [46]—the decline in drift opportunity with increased coverage (upper panels, Fig 4) is slower than that predicted for the attack rate (Fig 2). As a consequence drift opportunity is barely reduced even for full population coverage with a lower efficacy vaccine (*ϵ*_v_ = 0.75) for all scenarios, and remains approximately equal to that for an unimpeded pandemic in scenario B when *ϵ*_v_ ≳ 0.5. Assuming a boosting vaccine and no strain-specific humoral immunity (scenario C), 50 per cent coverage with a vaccine reducing the infectiousness of naïve hosts by *ϵ*_v_ = 0.25 reduces the drift opportunity to approximately 75 per cent of that for an unimpeded epidemic (Fig 4). Drift opportunity is minimised when vaccines are distributed to naïve hosts, mirroring the trends seen in the attack rate (left panel, Fig 3).

**Fig 5:**
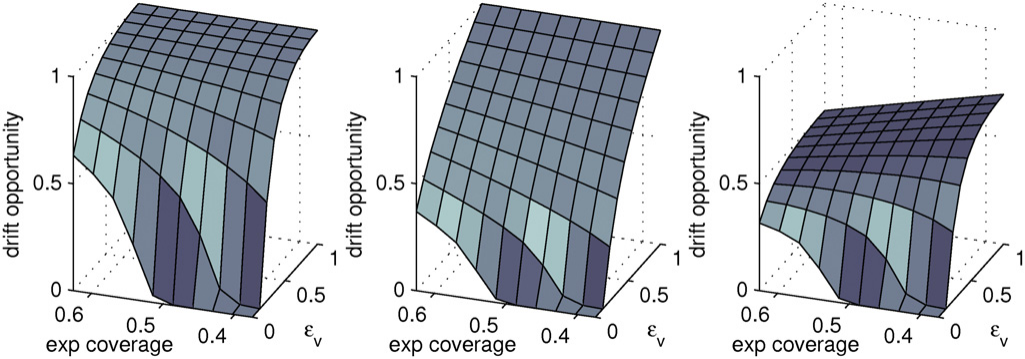
Drift opportunity in scenario C. Drift opportunity for scenario C with targeted distribution of a vaccine stockpile sufficient for 50 per cent coverage and assuming that pre-vaccination *R*_eff_ = 2. The axis labelled *ϵ*_v_ indicates the relative infectiousness of vaccinated and unvaccinated naïve hosts and “exp coverage” indicates the fractional vaccine coverage in experienced hosts (vaccine distribution is random when exp coverage is 0.5). Each panel corresponds to a different assumption regarding the form for the per infection rate of antigenic emergence *g*_0,*i*_; equal probability per infection (*left*), proportional to host infectiousness (*middle*), and zero for vaccinated hosts with existing CTL protection (*right*). The vertical axis show the probability of emergence *P*_emg_ of an antigenically novel variant over the course of the epidemic relative to the same scenario without intervention.

We focus on exploring variations in the achievable reduction in drift opportunity with the choice of *g*_0,*i*_ for fixed vaccine coverage of 50 per cent in scenario C with homogeneous mixing (Fig 5). Reductions in drift opportunity are naturally larger when the per infection probability of emergence of antigenic variants scales with host infectiousness (*g*_0,*i*_ = *ϵ*_*i*_) than if this per infection rate is uniform (*g*_0,*i*_ = 1). General population vaccination achieving 50 per cent coverage (*i.e.* exp coverage = 0.5) using a vaccine with *ϵ*_v_ = 0.25 can reduce drift opportunity to approximately 47 per cent of that for an unimpeded epidemic in this situation (see Fig 5). As for the attack rate, drift opportunity is minimised when vaccines are distributed to naïve hosts, despite the very low infectiousness of vaccinated experienced hosts when the vaccine has a boosting action.

If CTL vaccination completely suppresses the per infection probability of emergence of variants from vaccinated experienced hosts (*i.e.* *g*_0,4_ = 0, *g*_0,*i*_ = 1 otherwise), then the drift opportunity is further reduced compared to the scenario where *g*_0,*i*_ = *ϵ*_*i*_ for *ϵ*_v_ ≳ 0.2 (Fig 5). In contrast to the first two forms for *g*_0,*i*_ discussed, distributing CTL-inducing vaccines to experienced hosts minimises the drift opportunity in scenario C when *ϵ*_v_ ≳ 0.25 (Fig 5). In particular, the drift opportunity is approximately 20 percentage points lower when a stockpile allowing 50 per cent coverage for *ϵ*_v_ = 0.75 is preferentially administered to experienced rather than naïve hosts. In contrast the attack rate is only 4 percentage points lower (69 per cent compared to 73 per cent) if naïve hosts rather than experienced hosts are targeted. If *ϵ*_v_ ⪅ 0.25 the drift opportunity remains more efficiently suppressed by targeting naïve hosts to curtail the epidemic size. This trend holds for our assumed choice of assortative mixing (data not shown). Targeting experienced hosts has similar benefits for suppressing drift opportunity for moderate to high values of *ϵ*_v_ in scenario B, but has muted benefit in scenario D due to the smaller contribution of experienced hosts to the overall attack rate (data not shown). Note that for the final choice of *g*_0,*i*_ presented (right panel, Fig 5) we have assumed that even vaccines that don’t reduce overall host infectiousness (*ϵ*_v_ = 1) can completely suppress the emergence of new variants in experienced hosts. In practice a threshold vaccine efficacy may be required to generate such an effect.

Our estimate for the selective advantage of an antigenically novel variant circulating at the conclusion of the first pandemic wave *R*_eff, new_/*R*_eff_ depends on both pre-pandemic immunity and the pandemic attack rate, and thus varies between scenarios and with vaccine coverage (lower panels, Fig 4). In the absence of pre-pandemic neutralising cross-reactive antibody (scenarios A–C), if a vaccination campaign is able to mitigate spread the relative effective reproduction ratios of the (assumed equally transmissible) antigenically novel variant and resident pandemic strain remains unity. If vaccination does not mitigate the pandemic, antigenically novel variants have a distinct advantage that increases with the pandemic attack rate. Although attack rates—and thus the size of the population with acquired immunity—are smaller when there is pre-pandemic antibody mediated immunity in scenario D, the advantage of antigenically novel variants is compounded by this pre-existing immunity. If antigenic escape were to occur in this scenario the new variant will likely displace the original pandemic strain (*R*_eff,new_/*R*_eff_ > 2), potentially triggering a second wave of severe infection unconstrained by the cross-reactive antibody that limited the initial wave. If a CTL-inducing vaccine offers truly heterologous protection that suppresses hosts potential to transmit all IAV strains equally, increasing vaccination coverage cannot erase the advantage afforded to antigenically novel strains that emerge in the presence of immunity-driven selective pressures unless they halt almost all transmission thereby rendering *P*_emg_ negligible (see also [46]). However, unlike traditional vaccination campaigns which would increase *R*_eff,new_/*R*_eff_ even if the pandemic were mitigated, CTL-inducing vaccines do not further enhance the selective pressure for new antigenic variants.

### Sensitivity analysis

In the above sections we have explored the sensitivity of expected vaccine impact—as measured by the attack rate and/or drift opportunity—on underlying population immunity, population mixing characteristics and vaccine action. Here we additionally consider the sensitivity of our results to the transmissibility of the pandemic virus, the period of vaccine distribution, and alternate profiles for pre-pandemic population immunity for the homogeneous mixing case.

#### Comparison of scenarios with fixed attack rate

In our primary analysis we considered a fixed initial growth rate (*R*_eff_), resulting in baseline epidemics with considerably smaller attack rates for scenario D due to the presence of sterilising antibodies reducing the size of the susceptible pool. If we instead assume a fixed baseline *attack rate* across scenarios (which requires decreasing the initial value of *R*_eff_ for scenarios A–C), the additional coverage required to halt an epidemic in scenario D relative to scenarios A–C is even more pronounced than for the fixed initial *R*_eff_ scenarios (S5 Fig). Similarly, the inflated potential for drift opportunity and selection of antigenic variants in scenario D (Fig 4) is greater when compared to scenarios A–C with the same attack rate (S6 Fig).

#### Timeline for vaccine distribution in moderate and severe epidemics

Increasing vaccine coverage slows the rate of transmission, which while reducing the attack rate, also prolongs the epidemic. The higher probability of generating new variants later in an epidemic in Equation (2) signals that increased vaccine coverage may drive an *increase* in drift opportunity in our model. This effect is most likely to be observed when the per infection probability of generating variants is uniform (*g*_0,*i*_ = 1). This phenomenon is not observed for our nominal choice of *R*_eff_ = 2 (see Fig 4). However an increase in drift opportunity relative to a scenario without vaccination is observed for more severe epidemics with *R*_eff_ ≳ 3 that unimpeded would resolve very quickly (see S8 Fig for an example of a severe epidemic for scenario C). As our drift opportunity is uncalibrated against true risk of antigenic change, it is difficult to assign a significance to fractional increases in its value, however the selective advantage of an antigenically distinct variant (as measured by the ratio of effective reproduction numbers for the drifted and resident strains) is particularly high for scenarios with higher *R*_eff_ due to the large number of people with infection-acquired immunity to the resident strain when the first wave is resolved (lower panels, S8 Fig). Trends in drift opportunity and *R*_eff_ ratios are similar for scenarios A, B, D with *R*_eff_ = 4 (data not shown).

We have thus far assumed vaccines are distributed rapidly upon emergence of the pandemic (at a constant rate over 10 days) when exploring the impact of vaccine coverage. However the impact of vaccination campaigns on attack rates and epidemic duration is naturally less pronounced when vaccines are distributed over a longer-timescale. Focusing on scenario C, we show the effect of increasing the duration of vaccine distribution for *R*_eff_ = 2, 4 in S7 and S8 Figs. Prolonged interventions that distribute vaccines over a period of 6 months have limited impact on the attack rate (even for lower initial *R*_eff_) highlighting the value of rapid or pre-emptive vaccine distribution. As epidemics are resolved more rapidly when vaccination does not immediately reduce the effective reproduction number, the enhancement of drift opportunity with increased coverage seen for a severe scenario is diluted when vaccine distribution is prolonged (S8 Fig).

#### Population pre-pandemic immune profile

If population mixing is assumed to be homogeneous, we can decouple the concepts of youth and naïvety in our model, and freely vary the experienced proportion *f*_*E*_. Values of *f*_*E*_ less than our baseline value of 0.8 may, for example, reflect population immunity if late effector cytokine secreting T cells found to correlate with reduced viral shedding [17] are rare and/or effective T cell immunity is short-lived (perhaps requiring highly elevated numbers of effector T cells [20]).

For scenarios A and C (which both assume that vaccines scale the infectiousness of all hosts by a constant factor *ϵ*_v_), attack rates for fixed initial *R*_eff_ are independent of the prevalence (*f*_*E*_) and strength (*ϵ*) of suppressed infectiousness due to cellular immunity acquired from pre-pandemic seasonal IAV infection(s). If vaccine action is saturating, or doesn’t scale with pre-pandemic host CTL immunity in another fashion, characterising the landscape of underlying cellular immunity (determined by *f*_*E*_ and *ϵ* in our model) is crucial for predicting the impact of a vaccine on the attack rate. S9 Fig illustrates the sensitivity of the impact of vaccination campaigns with fixed coverage (50 or 75 per cent) on the values of *ϵ*, *f*_*E*_ and *ϵ*_v_; as *ϵ*_v_ increases the benefit of vaccination campaigns becomes increasingly dependent on having a low fraction of experienced hosts (low *f*_*E*_) and/or poor immunity amongst these hosts (high *ϵ*).

In scenario D, the unimpeded attack rate is strongly constrained by the fraction of experienced hosts with sterilising antibodies. However, the attack rate in the presence of an intervention in which vaccines are distributed to the general population with fixed coverage has only weak dependence on the proportion with sterilising antibodies, and minimal/no dependence on the strength of the underlying cellular immunity *ϵ* (see S10 Fig). This result reiterates the inefficiency of general population CTL-inducing vaccine roll-out when there is pre-pandemic antibody-mediated immunity.

## Discussion

We have explored a set of pandemic scenarios with differing underlying cellular and humoral immunity and considered the impact of two classes of CTL-inducing vaccines (saturating or boosting) motivated by potential CTL-inducing vaccine candidates. Our analysis is novel in its aims to capture the current immunological understanding of cellular immunity in population-level IAV pandemic transmission models. Doing so enables us to highlight the uncertainties in predicting the impact of wide-spread use of CTL-inducing vaccines against a pandemic virus and provide a valuable reference for informing the performance requirements of high impact CTL-inducing vaccines.

We have focused on the pandemic attack rate and drift opportunity as proxies for the impact of an intervention with CTL-inducing vaccines. By fixing the initial pandemic growth rate but altering the underlying population immune profile, we have shown that if using a boosting vaccine (scenarios A, B, D), the anticipated attack rate of a pandemic outbreak depends strongly on the extent of sterilising cross-reactive antibody in influenza-experienced (*i.e.* effectively assumed to be older) sub-populations, but not the strength and extent of cross-protective cellular immunity acting only on host infectiousness (Fig 2 and S10 Fig). When sterilising cross-reactive antibody is common amongst influenza-experienced hosts (scenario D), the high force of infection rapidly depletes the most infectious and susceptible hosts, but the epidemic attack rate is lower (Fig 2). Such trends are consistent with the observations of higher attack rates amongst younger hosts in the first wave of pH1N12009 [77, 78], although age-dependent mixing rates [61] in addition to the presence of pre-pandemic antibody in older hosts were likely responsible for this trend. In our hypothetical scenario akin to the emergence of a virus like H3N2v (scenario D), general population distribution of CTL-inducing vaccines is a particularly inefficient way to reduce the attack rate. If using a saturating vaccine (scenario B), or similar vaccine in which the reduction in infectiousness of vaccinated hosts depends on their pre-pandemic immunity, the mitigating impact depends crucially upon the strength and prevalence of pre-existing cellular immunity. If pre-existing cellular immunity is common (*i.e.* large *f*_*E*_), effective (*i*.*e*. *ϵ* < 1), and difficult to boost with vaccines (*i.e.* moderate *ϵ*_v_) as explored in scenario B, general population vaccination campaigns will be inefficient at controlling the spread of an emerging pandemic virus.

We have highlighted the benefit of targeted distribution strategies for CTL-inducing vaccines for minimising attack rates across all scenarios in which there is heterogeneity in pre-pandemic CTL mediated immunity (scenarios B–D). Vaccinating influenza-naïve hosts most likely to transmit has the largest impact on the attack rate, even when there are no other differences in the immune status or mixing behaviour of naïve and experienced hosts. If influenza-naïve hosts are typically children, targeting such sub-populations is likely to be of enhanced benefit for controlling the overall outbreak when taking into account their increased mixing intensity (S4 Fig). If a sub-population of influenza-experienced hosts have neutralising cross-reactive antibody to the pandemic virus (scenario D), moderately efficacious vaccines are more likely to mitigate a pandemic when targeted toward younger/naïve hosts than in other scenarios with the same initial growth rate *R*_eff_ (S4 Fig).

We have demonstrated that depending on the underlying population immunity and vaccine action, vaccination campaigns may have very different impact on the opportunity for the pandemic virus to undergo antigenic change over the pandemic season, for fixed initial growth rate *R*_eff_. If the per infection probability of emergence of antigenic variants is uniform across the population, drift opportunity is difficult to suppress unless the pandemic can be mitigated. If there is little pre-existing CTL-mediated immunity or the vaccine is able to boost this further in most hosts (scenarios A and C), then extensive CTL-inducing vaccination campaigns may significantly decrease the attack rate and modestly reduce the opportunity for antigenic change. In such scenarios prolonged use of a CTL-inducing vaccine would likely modestly slow the rate of emergence of antigenically novel clusters [46]. However, if pre-pandemic CTL mediated immunity is common, and the vaccine cannot always boost this further (scenario B), vaccination may have little impact on the attack rate and negligible impact on the ability of the virus to undergo antigenic change.

Uncertainties in the role of enhanced CTL responses in altering the within-host generation and selection of antigenic variants further cloud expectations for the long-term role of CTL-inducing vaccines on antigenic evolution. If the per infection probability of generating new variants (*g*_0,*i*_) scales with host infectiousness and/or the rapidity of viral clearance, the probability of antigenic replacement may decrease more rapidly with coverage than predicted by Arinaminpathy *et al.* [46]. If CTL-inducing vaccination facilitates rapid clearance in primed adults, such that vaccinated adults do not contribute to the drift opportunity, targeting adults may be optimal for controlling antigenic drift in situations where the vaccine efficacy and achievable coverage is such that the pandemic cannot be mitigated.

### Study limitations

We have adopted a minimal model to explore the impact of prior immunity and CTL-vaccine action on CTL-inducing vaccination campaign impact. This choice allowed us to explore a broader set of pandemic and vaccination scenarios, including the possibility of heterogeneous pre-pandemic cellular immunity, than explored in previous work [46]. As we model the transmission of a single pandemic IAV strain, and include within-host effects of CTL immunity through stratification of host infectious type, we can only estimate the potential for antigenic drift and strain replacement after a single epidemic wave without assigning this potential a calibrated probability. Population-level IAV circulation may also be influenced by within-host and population mixing effects not considered in our model: Luo *et al.* suggest that partial protection, by preventing target cell depletion, increases the opportunity for novel viruses to attain higher within-host viral loads [79], and the pattern of immune responses in chains of infection can also influence viral selection [80]. Our model assumes a simple distribution of pre-pandemic cellular and humoral immunity, which likely does not capture all age-cohort dependent trends in immune status. However detailed models for age-structured T cell immunity are difficult to justify given the scarcity of available data on human T cell immunity.

### Implications

Our modelling demonstrates that the utility of CTL-inducing vaccine candidates is not guaranteed, and provides insight into the efficacy and distribution strategies required to control pandemic influenza with next-generation CTL-inducing vaccines. In particular we have demonstrated that vaccine impact may hinge on as yet uncharacterised cellular immunity, with vaccination campaigns less likely to be of use if this is common and difficult to boost. However even if CTL-mediated immunity amongst adults is common and can be enhanced with vaccination, the most efficient way to control future pandemics may be to boost CTL immunity in younger hosts, regardless of considerations of enhanced transmission due to contact patterns that result in recommendations of preferential vaccination of children with traditional vaccines [76, 81]. Focusing on developing CTL-inducing vaccines that induce protective responses in hosts with naïve T cell pools may therefore be of particular importance. Only if a CTL-inducing vaccine performs particularly poorly in naïve/unprimed hosts might vaccination campaigns targeting experienced hosts be preferable for reducing the attack rate. Vaccination campaigns that aim to prime naïve T cells will likely have ongoing benefit—enhancing post-pandemic cellular immune responses to all IAV—even if the initial protective immune response triggered by the vaccine wanes over time [20]. However the prevention of senescence of T cell immunity might offer alternative motivation for administering CTL-inducing vaccines to older hosts [43].

We have explored the extent to which the genealogy of the pandemic strain may also limit the impact of CTL-inducing vaccination campaigns. A key determinant of the impact of CTL vaccination campaigns, particularly those aiming to vaccinate adults, will be the coupling of cohorts with boostable CTLs and neutralising antibody. Most human IAVs have their origin in avian variants [82], but viruses with the appropriate receptor specificity are more likely cross-over from swine, and IAV subtypes that circulate widely in swine are also those that circulate as seasonal influenza in humans [83]. As illustrated by the emergence of H3N2v, IAVs at the human-swine interface may be antigenically similar to recently circulating human seasonal IAV, and induce neutralising cross-reactive antibody in many adults that likely supersedes the protection afforded by enhanced CTL responses. Cellular immunity—which likely cannot prevent infection but only suppress transmission—may be of limited relevance for controlling the spread of such a virus. The presence of other heterologous immune responses to the pandemic virus, such as cross-reactive antibodies to sub-dominant epitopes [84], are likewise of lesser consequence if there is sterilising immunity to the immunodominant epitopes. Enhancing cellular immunity is more likely to aid pandemic control when the pandemic virus has an antigenic subtype that has experienced limited circulation in humans (scenarios A–C), such as the avian viruses H5N1 IAV [85] or the recently emerged H7N9 IAV [52]. These insights add to understanding of the role of pre-pandemic cellular and humoral immunity—in concert with host mixing effects—in determining the risk landscape for infection with pandemic viruses.

The influence of CTL-mediated immunity on host propensity to transmit new genetic variants may be an important consideration when designing strategies to minimise the risk of viral drift during a pandemic [67, 68]. We have demonstrated that the influence of CTL-inducing vaccination campaigns on antigenic drift may be greater than suggested by previous modelling work [46]. In contrast to the benefits of targeting children to reduce the attack rate, if CTL-inducing vaccines facilitate rapid clearance in primed hosts, targeting adults may be an efficient way to reduce the drift opportunity when the vaccination campaign does not have the power to significantly curtail the attack rate.

### Open questions

There is much scope to enhance our understanding of CTL-immunity and the likely utility of CTL-inducing vaccines by developing mathematical transmission models that incorporate the accumulating insight from immunological and epidemiological studies of the role of cellular immunity in regulating IAV infection in humans. Comprehensive modelling of CTL vaccination impact will require more detailed characterisation of the development and maintenance of memory CTL, the role of memory CTL on influenza infection, and the distribution of this immunity in socially realistic populations. Together with further study of influenza T-cell immunology in animal models, insights from household-based cohort studies or challenge studies will be key for modelling the development and prevalence of functional T cell immunity. Within-host modelling of IAV infection in the presence of CTL responses that can reproduce emerging experimental data will be required to aid understanding of the potential for CTL-inducing vaccines to alter the likelihood that hosts transmit IAV variants. Modelling of IAV circulation and evolution over time-scales longer than a single season that accounts for the development and maintenance of influenza-specific memory CTL will be required to predict the impact of sustained wide-spread CTL-inducing vaccine usage, including the risks and consequences of CTL vaccine escape.

Our results highlight the need for vaccine design to attend to our developing understanding of the ontology and evolution of T cell immunity in humans (e.g. [86]). Epidemiological studies looking for exposure dependent differences between precursor and effector CTL pools, will give insight into whether CTL-inducing vaccines might alter this response to boost ‘sub-dominant’ epitope-specific CTLs and provide protection superior to that triggered by natural infection (akin to scenario C), providing insight into the optimal design and potential efficacy of CTL-inducing vaccines against IAV.

Consideration of the utility of multi-faceted public health responses that use CTL-inducing vaccines to buy time before a strain-specific vaccine can be distributed, and/or use of CTL-inducing vaccines in tangent with traditional anti-viral drugs, are also of interest. In future work we will explore strategies for the optimal use of CTL-inducing vaccines that take into account sociologically important differences in mixing behaviour for adults and children in addition to differences in immunological memory to IAVs.

## Supporting Information

S1 Fig Sub-population attack rates with assortative mixing. Predicted attack rate as a function of general population vaccination coverage for scenarios A–D (left to right) assuming *R*_eff_ = 2. Red lines indicate attack rates for naïve hosts, blue lines for experienced hosts, and black lines indicate population-average values.
(EPS)

S2 Fig Sub-population attack rates in scenario D. Predicted attack rate as a function of general population coverage for scenario D assuming *R*_eff_ = 2 and random (left) or weakly assortative mixing (right) as described in the main text. As for the figures in the main text, solid lines indicate *ϵ*_v_ = 0.25, dashed lines *ϵ*_v_ = 0.5 and dotted lines *ϵ*_v_ = 0.75. Red lines indicate attack rates for naïve hosts, blue lines for experienced hosts, and black lines indicate population-average values. The grey lines show the results for scenario C with the same initial effective reproduction number and homogenous mixing.
(EPS)

S3 Fig Attack rates for targeted vaccination with assortative mixing. Epidemic attack rates for targeted distribution of a vaccine stockpile enabling 50 per cent population coverage assuming weakly assortative mixing (as described in the main text) and *R*_eff_ = 2 in scenarios B (*left*), C (*middle*) and D (*right*). The axis “exp coverage” indicates the fractional coverage in influenza-experienced hosts, with exp coverage = 0.5 corresponding to general population distribution of vaccines.
(EPS)

S4 Fig Reductions in attack rate for targeted vaccination campaigns. Absolute decrease in the attack rate (coded by the coloured legend) compared to a like scenario (with the same *ϵ*_v_ and 50 per cent coverage) where influenza-experienced hosts are preferentially targeted (*i.e.* exp coverage equal to 5/8) for scenarios B–D. *Upper* panels correspond to assumptions of homogeneous mixing and *lower* panels for weakly assortative mixing as described in the main text. Note that this figure is an alternate representation of the results in Fig 3. An optimal value for *ϵ*v for each set of scenarios arises due to the benefit of redirecting an imperfect vaccine to the sub-population more likely to transmit infections.
(EPS)

S5 Fig Attack rate with coverage for fixed baseline attack rate scenarios. Predicted attack rates for four different pandemic and vaccine action scenarios as a function of general population coverage with a CTL-inducing vaccine. Baseline attack rates (rather than initial values of *R*_eff_) have been *fixed* at ∼ 48 per cent for all scenarios. In each panel the solid line indicates *ϵ*_v_ = 0.25, with dashed and dotted lines corresponding to *ϵ*_v_ = 0.5 and 0.75 respectively. Scenario A captures a population without pre-existing CTL mediated immunity to the pandemic strain that experiences uniform vaccine efficacy. In scenarios B and C, 80 per cent of the population have CTL-mediated immunity reducing infectiousness by 50 per cent, with vaccine action saturating and boosting respectively. Scenario D assumes that 50 per cent of those with naturally acquired CTL immunity also have cross-reactive protective antibody and vaccine action is boosting.
(EPS)

S6 Fig Drift opportunity with coverage for fixed baseline attack rate scenarios. **Upper panels:** Drift opportunity as a function of coverage for scenarios A–D with *fixed baseline attack rate* of ∼ 48 per cent across all scenarios (rather than fixed initial *R*_eff_) and *g*_0,*i*_ = 1. **Lower panels:** The ratio of effective reproduction numbers of antigenically drifted and pandemic variants. In each panel the solid line indicates the relative infectiousness in vaccinated naïve and naïve hosts is *ϵ*_v_ = 0.25, with dashed and dotted lines *ϵ*_v_ = 0.5 and 0.75 respectively.
(EPS)

S7 Fig Impact of vaccination campaign duration in moderate scenarios. Predicted attack rate (*upper* panels) and drift opportunity (*lower* panels) as a function of general population vaccine coverage for Scenario C with an initial *R*_eff_ = 2 and vaccines distributed over 10 days (*i.e.* essentially pre-emptive vaccination, *left*), 90 days (*middle*) or 182 days (*right*). As for the figures in the main text, solid lines indicate *ϵ*_v_ = 0.25, dashed lines *ϵ*_v_ = 0.5 and dotted lines *ϵ*_v_ = 0.75.
(EPS)

S8 Fig Impact of vaccination campaign duration in severe scenarios. Predicted attack rate (*upper* panels), drift opportunity (*middle* panels) and ratio of effective reproduction numbers of drifted and resident strains (*lower* panels) as a function of general population vaccine coverage for Scenario C with an initial *R*_eff_ = 4 and vaccines distributed over 10 days (*left* panels), 90 days (*middle* panels) or 182 days (*right* panels). As for the figures in the main text, solid lines indicate *ϵ*_v_ = 0.25, dashed lines *ϵ*_v_ = 0.5 and dotted lines *ϵ*_v_ = 0.75. Note the differences in the scales of the y-axes when compared to S7 Fig.
(EPS)

S9 Fig Impact of pre-pandemic immunity: varations on scenario B. **Upper panels:** Attack rate assuming 50 per cent coverage of a saturating vaccine in a homogeneously mixing population as a function of the fraction of influenza experienced hosts *f*_*E*_ and suppression of infectiousness of unvaccinated experienced hosts *ϵ*. **Lower panels:** Attack rate assuming 75 per cent coverage of a saturating vaccine in a homogeneously mixing population as a function of the fraction of influenza-experienced hosts *f*_*E*_ and suppression of infectiousness of unvaccinated experienced hosts *ϵ*. Parameters corresponding to scenario B are marked in each panel by a red dot.
(EPS)

S10 Fig Impact of pre-pandemic immunity: variations on scenario D. Attack rate assuming *f*_*E*_ = 0.8 (as for the main results section) a function of the strength of pre-pandemic cellular immunity *ϵ* and the proportion of influenza-experienced hosts with humoral immunity (“fraction of *f*_*E*_ with Abs”). Results are shown for an unimpeded epidemic (*l*eft), assuming 50 per cent general population coverage with a boosting vaccine that suppresses the infectiousness of vaccinated naïve hosts by *ϵ*_v_ = 0.25 (*m*iddle) and as for the middle panel but assuming 75 per cent general population coverage (*r*ight). Parameter values corresponding to scenarios C and D are marked in each panel by red and blue dots respectively.
(EPS)
